# Reduced *Clostridium difficile* Tests and Laboratory-Identified Events With a Computerized Clinical Decision Support Tool and Financial Incentive

**DOI:** 10.1017/ice.2018.53

**Published:** 2018-04-12

**Authors:** Gregory R. Madden, Ian German Mesner, Heather L. Cox, Amy J. Mathers, Jason A. Lyman, Costi D. Sifri, Kyle B. Enfield

**Affiliations:** 1 Division of Infectious Diseases and International Health, Department of Medicine, University of Virginia Health System, Charlottesville, Virginia; 2 Health Information and Technology, University of Virginia Health System, Charlottesville, Virginia; 3 Department of Pharmacy Services, University of Virginia Health System, Charlottesville, Virginia; 4 Clinical Microbiology Laboratory, Department of Pathology, University of Virginia Health System, Charlottesville, Virginia; 5 Department of Public Health Sciences, University of Virginia Health System, Charlottesville, Virginia; 6 Office of Hospital Epidemiology/Infection Prevention and Control, University of Virginia Health System, Charlottesville, Virginia; 7 Division of Pulmonary and Critical Care Medicine, Department of Medicine, University of Virginia Health System, Charlottesville, Virginia; 8 Office of Quality and Performance Improvement, University of Virginia Health System, Charlottesville, Virginia.

## Abstract

We hypothesized that a computerized clinical decision support tool for *Clostridium difficile* testing would reduce unnecessary inpatient tests, resulting in fewer laboratory-identified events. Census-adjusted interrupted time-series analyses demonstrated significant reductions of 41% fewer tests and 31% fewer hospital-onset *C. difficile* infection laboratory-identified events following this intervention.

*Infect Control Hosp Epidemiol* 2018;39:737–740

*Clostridium difficile* is the most common causative pathogen of healthcare-associated infection (HAI) in the United States, resulting in significant cost, morbidity, and mortality.^1^ Because many hospitals have adopted highly sensitive nucleic acid amplification testing (NAAT) for *C. difficile* in favor of less sensitive tests, overdiagnosis of *C. difficile* infection (CDI) is suspected to be common, and up to half of hospitalized patients with positive *C. difficile* NAAT may not need treatment.^2^ Positive tests in patients who are not infected may lead to overtreatment and increased healthcare costs. One explanation for CDI overdiagnosis is that some patients with low pretest probability for infection may be inappropriately tested. Identifying “true” disease is essential to optimizing management and avoiding overtreatment.

Improving test utilization through diagnostic stewardship, such as through computerized clinical decision support (CCDS), is a recognized means by which hospitals may attempt to reduce cost and diagnostic error.^3^ While some CCDS interventions improve provider performance and patient outcomes, others are ineffective.^4^ ^,^ ^5^ Here, we report the implementation of a CCDS tool coupled with education and trainee incentives to reduce *C. difficile* testing.

## METHODS

At a 619-bed tertiary-care hospital, we performed a quasi-experimental retrospective cohort study analyzing rates of inpatient *C. difficile* test ordering and National Healthcare Safety Network (NHSN)-defined hospital-onset (ie, occurring on hospital day >3) *C. difficile* infection (HO-CDI) laboratory-identified (LabID) events^6^ before and after the introduction of a CCDS tool with nurse and provider education along with a financial incentive for graduate medical education (GME) trainees. The CCDS tool was developed after internal auditing by antimicrobial stewardship identified that 10 of 15 HO-CDI events (67%) during a 1-month period potentially lacked an indication for testing.

**FIGURE 1:**
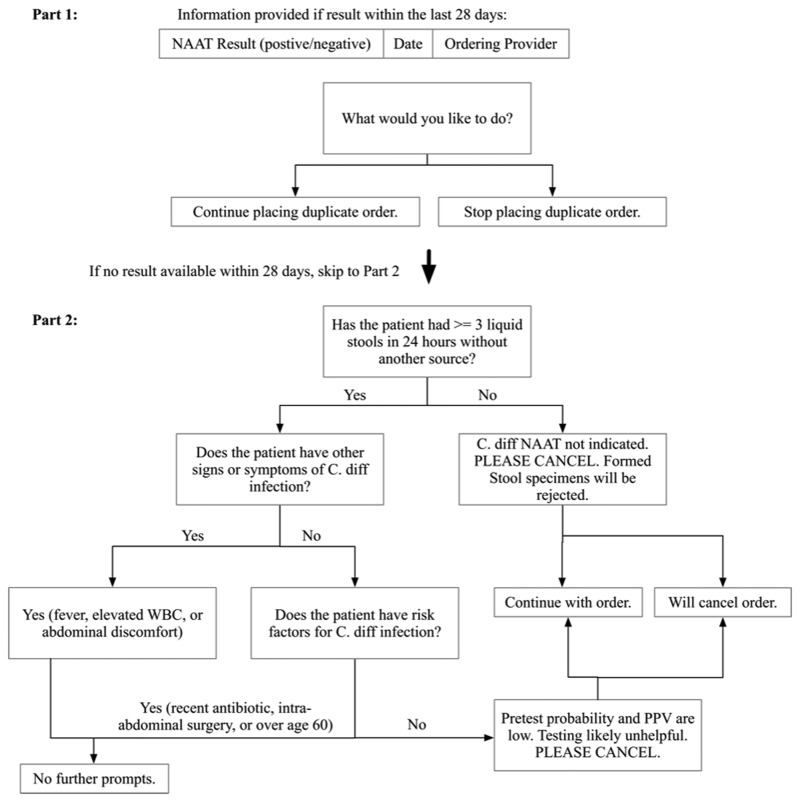
Two-part clinical decision support algorithm. NOTE. NAAT, nucleic acid amplification test for *Clostridium difficile*; C. diff, *C. difficile*; PPV, positive predictive value; WBC, white blood cell count.

The 2-part CCDS tool first displayed a duplicate-order information screen listing *C. difficile* test results within 28 days. Second, a series of questions designed to guide appropriate testing was presented to the ordering provider. The algorithm (Figure 1) was designed to highlight duplicate *C. difficile* tests that may be low yield^7^ and practice guidelines recommending testing only of symptomatic patients, while considering risk factors including antibiotic use, intra-abdominal surgery, and advanced age.^8^ A step-wise algorithm was chosen based on limitations of screen size and ease of reading (see Supplementary Material for software demonstration). A test could be ordered regardless of provider responses. According to the existing laboratory protocol, solid stool specimens were rejected for NAAT testing.

The CCDS tool was preceded by a series of educational efforts presented to all providers and nurses, including email, flyers, and a brief video (see Supplementary Material). These efforts explained the rationale for the CCDS, provided guidelines on appropriate *C. difficile* specimens, and demonstrated the tool. A representative body of GME trainees performed in-person training with house staff in each inpatient department. Education occurred over a 2-week period prior to activation of the CCDS tool with a reminder message on the day of implementation. In addition, GME trainees were provided a 0.8% bonus (jointly funded by the UVA Office of Graduate Medical Education and UVA Health System) at the end of the academic year (June 2017) if testing by GME providers fell by ≥25% compared to the preintervention period.

The CCDS tool was developed in response to a broad, multidisciplinary commitment to HO-CDI reduction endorsed by hospital leadership. Monitoring was conducted daily using an electronic *C. difficile* dashboard reflecting all daily tests, new positive tests, duplicate tests, and test attempts “prevented” by the CCDS, provided to hospital staff and administration with unit and service attributions. The antimicrobial stewardship team performed chart reviews of patients with positive tests, evaluating appropriate testing and other opportunities to reduce HO-CDI. In addition to CCDS implementation, peroxyacetic acid/hydrogen peroxide-based cleaner was adopted hospital-wide in May 2017 to replace quaternary ammonium and bleach for daily and terminal hospital room disinfection. In addition, a policy change on April 2017 restricted antibiotics for neurosurgical drain prophylaxis. No other major new *C. difficile*-related infection control interventions were implemented during the study period.

An 18-month preintervention control period (June 2014 to November 2016) was compared to a 10-month postintervention period (December 2016 to September 2017) following CCDS implementation on December 5, 2016. In this analysis, HO-CDI and test count data were normalized to monthly patient days. An order was considered prevented if providers initiated but did not complete a *C. difficile* NAAT order. Canceled test orders and samples not submitted to the laboratory were excluded from the analysis.

Testing rates and proportions of positive tests were compared between the intervention groups using independent sample *t* tests and the χ^2^ test, respectively. Due to fewer total HO-CDI events, a quasi-Poisson model was used to assess changes in HO-CDI counts between pre- and post intervention periods, using patient days as an offset. Analyses were performed using statistical R version 3.4.1 software (R Core Team, Vienna, Austria). The University of Virginia Internal Review Board approved this study (no. 20082).

## RESULTS

**FIGURE 2:**
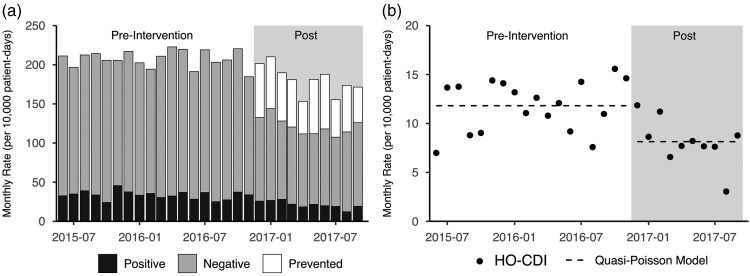
Monthly *C. difficile* tests and hospital-onset *C. difficile* infection (HO-CDI) laboratory-identified (LabID) events detected with CCDS tool pre- and postintervention. (a) Monthly rates of test results. (b) Trends of monthly HO-CDI rates over the same period. The dotted line depicts predicted values using the quasi-Poisson model.

A total of 233,577 and 132,641 patient days were observed during the pre- and postintervention periods, respectively. The CCDS tool was associated with a 41% reduction in the overall rate of *C. difficile* tests (208 results per 10,000 patient days preintervention compared to 122 per 10,000 patient days postintervention; *P*<.001). We also observed 31% fewer HO-CDI events (11.8 per 10,000 patient days preintervention versus 8.1 postintervention; *P*=.001) (Figure 2).

Duplicate negative results (defined as a negative result within 3 days following a previous negative) decreased from 5.7 per 10,000 patient days (134 duplicate negatives) preintervention to 1.5 per 10,000 patient days (20 duplicate negatives) postintervention (*P* < .001). Duplicate positive results (within 14 days following a previous positive test) decreased from 0.9 per 10,000 patient days (22 duplicate positives) preintervention to 0.15 per 10,000 patient days (2 duplicate positives) postintervention (*P*=.004). A nonsignificant reduction in laboratory-rejected stool specimens occurred, from 15.9 per 10,000 patient days (371 rejected) preintervention to 10.8 per 10,000 patient days (143 rejected) postintervention (*P*=.064). The percentage of positive *C. difficile* results did not significantly change from 16.2% preintervention to 17.5% postintervention (*P*=.195).

## DISCUSSION

In this quasi-experimental quality improvement study, CCDS targeting inappropriate *C. difficile* testing was associated with significantly reduced rates of overall tests performed. CCDS was also associated with reduced HO-CDI events. Duplicate results were significantly reduced postintervention, while the percentage of positive tests and the rate of specimens rejected by the laboratory were not significantly changed.

To our knowledge, this is the first report of a CCDS tool for *C difficile* testing for which prevented tests can be counted. Numbers of prevented tests do not appear to entirely account for reductions in total test numbers over time, implying a possible influence on ordering behavior.

Prior studies examining CCDS for addressing CDI have shown mixed results. One “best practice alert” for CDI treatment did not show improved compliance with treatment guidelines.^5^ Of 2 studies of CCDS for *C. difficile* testing that targeted laxative use (an issue not addressed in our CCDS tool), only 1 demonstrated reduced rates of total tests and *C. difficile* events.^9^ ^,^ ^10^ Nicholson et al^11^ observed reduced *C. difficile* testing rates with CCDS-based guidance in pediatric patients, but CDI rates were not compared.

The strengths of this study include its size and broad deployment of CCDS across an entire hospital system, contributing to its generalizability. However, the study has several limitations. As a quasi-experimental retrospective analysis, we could not control for potential time-varying confounders, and a 10-month postintervention period did not allow us to determine the long-term durability of our findings. The potential influences of institutional changes such as the monetary benefit of reduced *C. difficile* testing by the GME trainees, antimicrobial stewardship activities, peroxyacetic acid-based cleaning, and increased overall awareness of *C. difficile* could not be fully separated from that of the CCDS tool. Analyses of additional data following the incentive payment are needed to determine the role the incentive played in the behavioral change. However, post hoc subgroup analyses excluding the 7-month financial incentive period (December 2016 to June 2017) and the peroxyacetic acid period (May to September 2017) demonstrated similar effects on testing and HO-CDI (data not shown). Further studies engaging providers across multiple settings (community and hospitals), potentially with randomization and without financial rewards or other interventions, would be helpful in establishing the generalizability of our results.

The observed reduction in LabID events theoretically reflects the prevention of potentially false-positive test results (eg, representing colonization, not infection) through reduced inappropriate tests but could also reflect improved antimicrobial stewardship, environmental factors, or a reduction of appropriately ordered tests. Although it is possible that reduced *C. difficile* testing may have led to missed or delayed CDI diagnoses resulting in undertreatment and patient harm, increased CDI-related complications or deaths were not noted during the study period (data not shown). Similarly, other studies using CCDS for *C. difficile* testing have not been associated with poor outcomes.^10^ ^,^ ^11^ Nevertheless, these studies have not systematically addressed the potential for diagnostic stewardship to cause patient harm. Future diagnostic stewardship studies for *C. difficile* NAAT or other testing methodologies should ideally include outcome measures targeted to patients with prevented tests to determine clinical relevance and patient safety.^3^

Optimizing test utilization remains a critical component of quality healthcare delivery. Understanding how computerized order entry can be leveraged to drive behavior is important in improving appropriate testing.

## Supplementary material

For supplementary material accompanying this paper visit http://dx.doi.org/10.1017/ice.2018.53. click here to view supplementary material

## References

[1] Lessa FC, Mu Y, Bamberg WM, et al Burden of *Clostridium difficile* infection in the United States. N Engl J Med 2015;372:825–834. 25714160 10.1056/NEJMoa1408913 PMC10966662

[2] Polage CR, Gyorke CE, Kennedy MA, et al Overdiagnosis of *Clostridium difficile* infection in the molecular test era. JAMA Intern Med 2015;175:1792–1801. 26348734 10.1001/jamainternmed.2015.4114 PMC4948649

[3] Madden GR, Weinstein RA, Sifri CD. Diagnostic stewardship for healthcare-associated infections: opportunities and challenges to safely reducing test use. Infect Control Hosp Epidemiol 2018;39:214–218. 29331159 10.1017/ice.2017.278 PMC7053094

[4] Hunt DL, Haynes RB, Hanna SE, et al Effects of computer-based clinical decision support systems on physician performance and patient outcomes. JAMA 1998;280:1339. 9794315 10.1001/jama.280.15.1339

[5] Revolinski S. Implementation of a clinical decision support alert for the management of *Clostridium difficile* infection. Antibiotics (Basel) 2015;4:667–674. 27025646 10.3390/antibiotics4040667 PMC4790319

[6] CDC/NHSN surveillance definitions for specific types of infections. Centers for Disease Control and Prevention website. https://www.cdc.gov/nhsn/pdfs/pscmanual/17pscnosinfdef_current.pdf. Updated January 2017. Accessed April 26, 2017.

[7] Cardona DM, Rand KH. Evaluation of repeat *Clostridium difficile* enzyme immunoassay testing. J Clin Microbiol 2008;46:3686–3689. 18845820 10.1128/JCM.00931-08 PMC2576601

[8] Cohen SH, Gerding DN, Johnson S, et al Clinical practice guidelines for *Clostridium difficile* infection in adults: 2010 update by the Society for Healthcare Epidemiology of America (SHEA) and the Infectious Diseases Society of America (IDSA). Infect Control Hosp Epidemiol 2010;31:431–455. 20307191 10.1086/651706

[9] Drees M, Dressler R, Taylor K, et al Testing stewardship: a “hard stop” to reduce inappropriate *C. diff* testing. Open Forum Infect Dis 2017;4(Suppl 1):S1–S2.

[10] White DR, Hamilton KW, Pegues DA, et al The impact of a computerized clinical decision support tool on inappropriate *Clostridium difficile* testing. Infect Control Hosp Epidemiol 2017;38:1204–1208. 28760168 10.1017/ice.2017.161

[11] Nicholson MR, Freswick PN, Di Pentima MC, et al The use of a computerized provider order entry alert to decrease rates of *Clostridium difficile* testing in young pediatric patients. Infect Control Hosp Epidemiol 2017;38:542–546. 28219462 10.1017/ice.2017.16

